# 3-(2-Bromo­eth­oxy)-4-(4-bromo­phen­yl)furan-5(2*H*)-one

**DOI:** 10.1107/S1600536810044508

**Published:** 2010-11-06

**Authors:** Zhu-Ping Xiao, Wen-Bin Yan, Zhu-Xiang Liu, Li-hua Chen, Xiao-Chun Peng

**Affiliations:** a College of Chemistry & Chemical Engineering, Jishou University, Jishou 416000, People’s Republic of China

## Abstract

In the title compound, C_12_H_10_Br_2_O_3_, the dihedral angle between the furan-5(2*H*)-one ring and the benzene ring is 1.2 (3)°. Two intra­molecular C—H⋯O inter­actions occur in the mol­ecule, both of which generate *S*(6) rings. The bromo­ethyl fragment is disordered over two sets of sites in a 0.773 (8):0.227 (8) ratio. In the crystal, inversion dimers linked by pairs of C—H⋯π inter­actions occur.

## Related literature

For background to furan­ones, see: Bailly *et al.* (2008 ▶); Weber *et al.* (2005 ▶).
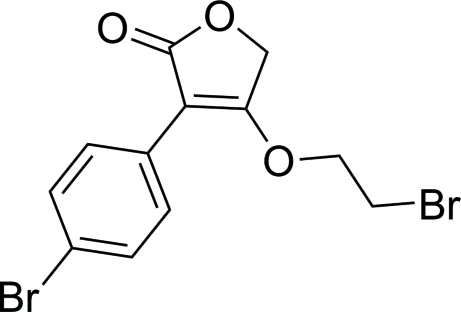


## Experimental

### 

#### Crystal data

C_12_H_10_Br_2_O_3_
*M* *_r_* = 362.02
Monoclinic, 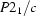

*a* = 8.6171 (13) Å
*b* = 10.4434 (16) Å
*c* = 13.958 (2) Å
β = 95.831 (3)°
*V* = 1249.6 (3) Å^3^
*Z* = 4
Mo *K*α radiation
μ = 6.48 mm^−1^
*T* = 298 K
0.20 × 0.10 × 0.10 mm

#### Data collection

Bruker SMART APEX CCD diffractometer
Absorption correction: multi-scan (*SADABS*; Sheldrick, 1996 ▶) *T*_min_ = 0.357, *T*_max_ = 0.564
7195 measured reflections
2582 independent reflections
1765 reflections with *I* > 2σ(*I*)
*R*_int_ = 0.025

#### Refinement

*R*[*F* ^2^ > 2σ(*F* ^2^)] = 0.061
*wR*(*F* ^2^) = 0.187
*S* = 1.05
2582 reflections
155 parameters
29 restraints
H-atom parameters constrained
Δρ_max_ = 1.37 e Å^−3^
Δρ_min_ = −1.15 e Å^−3^

### 

Data collection: *SMART* (Bruker, 2007 ▶); cell refinement: *SAINT* (Bruker, 2007 ▶); data reduction: *SAINT*; program(s) used to solve structure: *SHELXS97* (Sheldrick, 2008 ▶); program(s) used to refine structure: *SHELXL97* (Sheldrick, 2008 ▶); molecular graphics: *SHELXTL* (Sheldrick, 2008 ▶); software used to prepare material for publication: *SHELXL97*.

## Supplementary Material

Crystal structure: contains datablocks global, I. DOI: 10.1107/S1600536810044508/hb5720sup1.cif

Structure factors: contains datablocks I. DOI: 10.1107/S1600536810044508/hb5720Isup2.hkl

Additional supplementary materials:  crystallographic information; 3D view; checkCIF report

## Figures and Tables

**Table 1:** Hydrogen-bond geometry (Å, °) *Cg*1 is the centroid of the C1–C6 benzene ring.

*D*—H⋯*A*	*D*—H	H⋯*A*	*D*⋯*A*	*D*—H⋯*A*
C2—H2⋯O1	0.93	2.35	3.018 (10)	129
C6—H6⋯O3	0.93	2.25	2.916 (8)	128
C9—H9*B*⋯*Cg*1^i^	0.97	2.80	3.632 (9)	144

Symmetry code: (i) 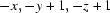.

**Fig. 1.**
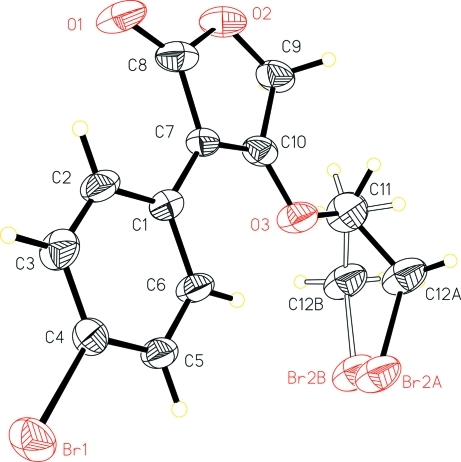
Molecular structure of the title compound, showing isplacement ellipsoids drawn at the 30% probability level.

**Fig. 2.**
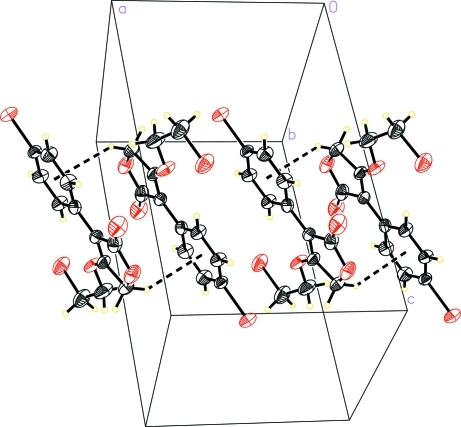
Dimers are formed through intermolecular C—H···π hydrogen bond pairs. Dashed lines indicate C—H···π contacts.

## supplementary crystallographic information

### Comment

Many compounds with γ-butyrolactone-core (furanone) show diverse biological activities such as antitumor and anti-inflammatory activity (Bailly *et al.*, 2008; Weber *et al.*, 2005). Recently, we focused our efforts to synthesize enamines with γ-butyrolactone-core for antibacterial activity screening. Herein, we report the crystal structure of the title compound (I).

Bond distance C7—C10 (1.334 (10) Å) is followed in the range of a typical double bond (1.32–1.38 Å), and the title compound was therefore identified as a furan-5(2*H*)-one not a furan-2(3*H*)-one. C10—O3 (1.348 (8) Å) bond has shorter bond distance than the standard C—O single bond (1.41–1.44 Å), but longer than C—O double bond (1.19–1.23 Å). This clearly indicated that an *sp*^3^ orbital of O3 is conjugated with the π molecular orbital of C7—C10 double bond, which was supported by the small torsion angle (0.4 (12) °) of C1—C7—C10—O3. The stereochemistry of the double bond in lactone ring was assigned as (*E*)-configuration based on X-ray crystallography of the title compound (Fig. 1). The butyrolactone moiety makes a dihedral angle of 1.2 (3) ° with the 4-fluorophenyl group. The side chain bromoethyl group is disorder (Fig. 1). C—H···Π contacts link molecules into dimers (Fig. 2), and the result dimers are packed by van der waals.

### Experimental

3-(4-Bromophenyl)-4-hydroxyfuran-5(2*H*)-one (0.77 g, 3 mmol) was added to a solution of 1,2-dibromoethane (2.8 g, 15 mmol) and triethylamine (0.7 g, 7 mmol) in dry acetone. The stirring was maintained at reflux temperature for 5 h. After the solvent was removed, the residue was partitioned between EtOAc and water. The organic layer was then dryed over MgSO4 and concentrated under reduced pressure. Flash chromatography (EtOAc/petroleum ether, 1/1, *v*/*v*) gave a fraction, which was partially evaporated to give the colorless blocks of (I).

### Refinement

All H atoms were placed in geometrically idealized positions and constrained to ride on their parent atoms with C—H = 0.93 and 0.96 Å for the aromatic and CH_2_ type H atoms, respectively. *U*_iso_ = 1.2*U*_eq_(parent atoms) were assigned for all H atoms.

### Crystal data

**Table:**

C_12_H_10_Br_2_O_3_	*F*(000) = 704
*M**_r_* = 362.02	*D*_x_ = 1.924 Mg m^−^^3^
Monoclinic, *P*2_1_/*c*	Mo *K*α radiation, λ = 0.71073 Å
Hall symbol: -P 2ybc	Cell parameters from 1832 reflections
*a* = 8.6171 (13) Å	θ = 2.6–26.1°
*b* = 10.4434 (16) Å	µ = 6.48 mm^−^^1^
*c* = 13.958 (2) Å	*T* = 298 K
β = 95.831 (3)°	Block, colorless
*V* = 1249.6 (3) Å^3^	0.20 × 0.10 × 0.10 mm
*Z* = 4	

### Data collection

**Table:**

Bruker SMART APEX CCD diffractometer	2582 independent reflections
Radiation source: fine-focus sealed tube	1765 reflections with *I* > 2σ(*I*)
graphite	*R*_int_ = 0.025
φ and ω scans	θ_max_ = 26.5°, θ_min_ = 2.4°
Absorption correction: multi-scan (*SADABS*; Sheldrick, 1996)	*h* = −10→10
*T*_min_ = 0.357, *T*_max_ = 0.564	*k* = −6→13
7195 measured reflections	*l* = −17→17

### Refinement

**Table:**

Refinement on *F*^2^	Primary atom site location: structure-invariant direct methods
Least-squares matrix: full	Secondary atom site location: difference Fourier map
*R*[*F*^2^ > 2σ(*F*^2^)] = 0.061	Hydrogen site location: inferred from neighbouring sites
*wR*(*F*^2^) = 0.187	H-atom parameters constrained
*S* = 1.05	*w* = 1/[σ^2^(*F*_o_^2^) + (0.1008*P*)^2^ + 2.0749*P*] where *P* = (*F*_o_^2^ + 2*F*_c_^2^)/3
2582 reflections	(Δ/σ)_max_ < 0.001
155 parameters	Δρ_max_ = 1.37 e Å^−^^3^
29 restraints	Δρ_min_ = −1.15 e Å^−^^3^

### Special details

**Table:**

Geometry. All e.s.d.'s (except the e.s.d. in the dihedral angle between two l.s. planes) are estimated using the full covariance matrix. The cell e.s.d.'s are taken into account individually in the estimation of e.s.d.'s in distances, angles and torsion angles; correlations between e.s.d.'s in cell parameters are only used when they are defined by crystal symmetry. An approximate (isotropic) treatment of cell e.s.d.'s is used for estimating e.s.d.'s involving l.s. planes.
Refinement. Refinement of *F*^2^ against ALL reflections. The weighted *R*-factor *wR* and goodness of fit *S* are based on *F*^2^, conventional *R*-factors *R* are based on *F*, with *F* set to zero for negative *F*^2^. The threshold expression of *F*^2^ > σ(*F*^2^) is used only for calculating *R*-factors(gt) *etc*. and is not relevant to the choice of reflections for refinement. *R*-factors based on *F*^2^ are statistically about twice as large as those based on *F*, and *R*- factors based on ALL data will be even larger.

### Fractional atomic coordinates and isotropic or equivalent isotropic displacement parameters (Å^2^)

**Table:**

	*x*	*y*	*z*	*U*_iso_*/*U*_eq_	Occ. (<1)
Br1	0.50480 (10)	0.34315 (8)	0.21873 (6)	0.0737 (4)	
Br2A	0.2920 (5)	0.92938 (17)	0.48473 (18)	0.0900 (8)	0.773 (8)
Br2B	0.2207 (13)	0.9329 (7)	0.4681 (7)	0.0900 (8)	0.227 (8)
C1	0.2553 (7)	0.4284 (6)	0.4943 (5)	0.0478 (14)	
C2	0.2843 (9)	0.3041 (7)	0.4667 (5)	0.0600 (18)	
H2	0.2534	0.2368	0.5041	0.072*	
C3	0.3561 (9)	0.2770 (7)	0.3870 (6)	0.0627 (18)	
H3	0.3711	0.1923	0.3696	0.075*	
C4	0.4069 (8)	0.3750 (7)	0.3319 (5)	0.0516 (15)	
C5	0.3815 (9)	0.5002 (6)	0.3576 (5)	0.0562 (17)	
H5	0.4147	0.5668	0.3205	0.067*	
C6	0.3076 (8)	0.5270 (6)	0.4379 (5)	0.0566 (17)	
H6	0.2920	0.6117	0.4549	0.068*	
C7	0.1745 (8)	0.4574 (7)	0.5800 (5)	0.0491 (15)	
C8	0.1126 (9)	0.3606 (9)	0.6426 (6)	0.067 (2)	
C9	0.0595 (10)	0.5553 (9)	0.7061 (5)	0.069 (2)	
H9A	0.1208	0.5923	0.7614	0.083*	
H9B	−0.0425	0.5954	0.6988	0.083*	
C10	0.1405 (8)	0.5708 (7)	0.6165 (5)	0.0548 (16)	
C11	0.1273 (12)	0.7904 (7)	0.6271 (7)	0.092 (3)	
H11A	0.1406	0.7743	0.6959	0.110*	0.773 (8)
H11B	0.0172	0.8050	0.6085	0.110*	0.773 (8)
H11C	0.2125	0.8202	0.6724	0.110*	0.227 (8)
H11D	0.0412	0.7671	0.6633	0.110*	0.227 (8)
C12A	0.2190 (15)	0.9106 (9)	0.6052 (6)	0.0900 (8)	0.773 (8)
H12A	0.3085	0.9160	0.6532	0.108*	0.773 (8)
H12B	0.1534	0.9840	0.6150	0.108*	0.773 (8)
C12B	0.076 (3)	0.899 (2)	0.5565 (19)	0.0900 (8)	0.227 (8)
H12C	0.0599	0.9763	0.5929	0.108*	0.227 (8)
H12D	−0.0235	0.8763	0.5214	0.108*	0.227 (8)
O1	0.1076 (8)	0.2448 (6)	0.6373 (5)	0.0886 (18)	
O2	0.0467 (7)	0.4217 (6)	0.7160 (4)	0.0808 (17)	
O3	0.1739 (7)	0.6861 (5)	0.5802 (4)	0.0731 (16)	

### Atomic displacement parameters (Å^2^)

**Table:**

	*U*^11^	*U*^22^	*U*^33^	*U*^12^	*U*^13^	*U*^23^
Br1	0.0856 (6)	0.0761 (6)	0.0639 (5)	0.0142 (4)	0.0292 (4)	−0.0115 (4)
Br2A	0.102 (2)	0.0635 (7)	0.1132 (11)	−0.0048 (10)	0.0551 (13)	−0.0016 (6)
Br2B	0.102 (2)	0.0635 (7)	0.1132 (11)	−0.0048 (10)	0.0551 (13)	−0.0016 (6)
C1	0.046 (3)	0.045 (4)	0.052 (3)	−0.003 (3)	0.008 (3)	0.007 (3)
C2	0.080 (5)	0.038 (4)	0.064 (4)	−0.003 (3)	0.016 (4)	0.012 (3)
C3	0.076 (5)	0.038 (4)	0.076 (5)	0.008 (3)	0.017 (4)	−0.002 (3)
C4	0.055 (4)	0.049 (4)	0.053 (3)	0.004 (3)	0.015 (3)	−0.008 (3)
C5	0.079 (5)	0.040 (4)	0.054 (4)	−0.005 (3)	0.024 (3)	0.002 (3)
C6	0.076 (5)	0.038 (3)	0.060 (4)	−0.004 (3)	0.028 (3)	0.006 (3)
C7	0.052 (4)	0.054 (4)	0.043 (3)	−0.005 (3)	0.012 (3)	0.005 (3)
C8	0.060 (4)	0.081 (6)	0.061 (4)	−0.002 (4)	0.015 (3)	0.022 (4)
C9	0.074 (5)	0.089 (6)	0.049 (4)	−0.004 (4)	0.026 (3)	0.000 (4)
C10	0.053 (4)	0.068 (5)	0.046 (3)	0.000 (3)	0.018 (3)	0.001 (3)
C11	0.116 (7)	0.071 (5)	0.096 (6)	−0.002 (5)	0.054 (5)	−0.024 (4)
C12A	0.102 (2)	0.0635 (7)	0.1132 (11)	−0.0048 (10)	0.0551 (13)	−0.0016 (6)
C12B	0.102 (2)	0.0635 (7)	0.1132 (11)	−0.0048 (10)	0.0551 (13)	−0.0016 (6)
O1	0.106 (5)	0.067 (4)	0.099 (4)	−0.010 (3)	0.037 (3)	0.031 (3)
O2	0.084 (4)	0.098 (5)	0.067 (3)	−0.003 (3)	0.039 (3)	0.021 (3)
O3	0.111 (4)	0.048 (3)	0.069 (3)	0.000 (3)	0.053 (3)	−0.006 (2)

### Geometric parameters (Å, °)

**Table:**

Br1—C4	1.895 (6)	C8—O2	1.377 (10)
Br2A—C12A	1.864 (5)	C9—O2	1.407 (11)
Br2B—C12B	1.878 (6)	C9—C10	1.501 (9)
C1—C2	1.384 (10)	C9—H9A	0.9700
C1—C6	1.397 (9)	C9—H9B	0.9700
C1—C7	1.476 (9)	C10—O3	1.348 (8)
C2—C3	1.357 (11)	C11—O3	1.352 (9)
C2—H2	0.9300	C11—C12A	1.531 (5)
C3—C4	1.378 (10)	C11—C12B	1.539 (6)
C3—H3	0.9300	C11—H11A	0.9700
C4—C5	1.379 (10)	C11—H11B	0.9700
C5—C6	1.372 (9)	C11—H11C	0.9700
C5—H5	0.9300	C11—H11D	0.9700
C6—H6	0.9300	C12A—H12A	0.9700
C7—C10	1.334 (10)	C12A—H12B	0.9700
C7—C8	1.471 (10)	C12B—H12C	0.9700
C8—O1	1.212 (10)	C12B—H12D	0.9700
			
C2—C1—C6	117.2 (6)	O3—C11—C12B	111.5 (12)
C2—C1—C7	122.0 (6)	C12A—C11—C12B	52.3 (13)
C6—C1—C7	120.8 (6)	O3—C11—H11A	109.1
C3—C2—C1	122.2 (6)	C12A—C11—H11A	109.1
C3—C2—H2	118.9	C12B—C11—H11A	139.4
C1—C2—H2	118.9	O3—C11—H11B	109.1
C2—C3—C4	120.0 (6)	C12A—C11—H11B	109.1
C2—C3—H3	120.0	C12B—C11—H11B	60.0
C4—C3—H3	120.0	H11A—C11—H11B	107.9
C3—C4—C5	119.4 (6)	O3—C11—H11C	109.3
C3—C4—Br1	121.9 (5)	C12A—C11—H11C	59.7
C5—C4—Br1	118.6 (5)	C12B—C11—H11C	109.3
C6—C5—C4	120.3 (6)	H11A—C11—H11C	53.5
C6—C5—H5	119.9	H11B—C11—H11C	141.2
C4—C5—H5	119.9	O3—C11—H11D	109.3
C5—C6—C1	120.8 (7)	C12A—C11—H11D	138.3
C5—C6—H6	119.6	C12B—C11—H11D	109.3
C1—C6—H6	119.6	H11A—C11—H11D	57.4
C10—C7—C8	106.0 (6)	H11B—C11—H11D	53.1
C10—C7—C1	129.2 (6)	H11C—C11—H11D	108.0
C8—C7—C1	124.8 (7)	C11—C12A—Br2A	119.6 (6)
O1—C8—O2	119.5 (7)	C11—C12A—H12A	107.4
O1—C8—C7	131.4 (8)	Br2A—C12A—H12A	107.4
O2—C8—C7	109.0 (7)	C11—C12A—H12B	107.4
O2—C9—C10	103.7 (6)	Br2A—C12A—H12B	107.4
O2—C9—H9A	111.0	H12A—C12A—H12B	106.9
C10—C9—H9A	111.0	C11—C12B—Br2B	113.0 (7)
O2—C9—H9B	111.0	C11—C12B—H12C	109.0
C10—C9—H9B	111.0	Br2B—C12B—H12C	109.0
H9A—C9—H9B	109.0	C11—C12B—H12D	109.0
C7—C10—O3	125.9 (6)	Br2B—C12B—H12D	109.0
C7—C10—C9	111.1 (6)	H12C—C12B—H12D	107.8
O3—C10—C9	123.0 (6)	C8—O2—C9	110.1 (6)
O3—C11—C12A	112.3 (6)	C10—O3—C11	116.9 (5)
			
C6—C1—C2—C3	−1.9 (11)	C8—C7—C10—O3	−178.9 (7)
C7—C1—C2—C3	178.8 (7)	C1—C7—C10—O3	0.4 (12)
C1—C2—C3—C4	1.8 (12)	C8—C7—C10—C9	1.8 (8)
C2—C3—C4—C5	−1.1 (11)	C1—C7—C10—C9	−178.9 (7)
C2—C3—C4—Br1	−178.9 (6)	O2—C9—C10—C7	−1.5 (9)
C3—C4—C5—C6	0.5 (11)	O2—C9—C10—O3	179.1 (7)
Br1—C4—C5—C6	178.4 (6)	O3—C11—C12A—Br2A	31.9 (14)
C4—C5—C6—C1	−0.6 (12)	C12B—C11—C12A—Br2A	−68.7 (13)
C2—C1—C6—C5	1.3 (11)	O3—C11—C12B—Br2B	−49 (2)
C7—C1—C6—C5	−179.4 (7)	C12A—C11—C12B—Br2B	53.0 (14)
C2—C1—C7—C10	178.8 (7)	O1—C8—O2—C9	−177.4 (8)
C6—C1—C7—C10	−0.5 (11)	C7—C8—O2—C9	0.4 (8)
C2—C1—C7—C8	−2.0 (11)	C10—C9—O2—C8	0.6 (8)
C6—C1—C7—C8	178.7 (7)	C7—C10—O3—C11	179.0 (8)
C10—C7—C8—O1	176.1 (9)	C9—C10—O3—C11	−1.7 (11)
C1—C7—C8—O1	−3.2 (13)	C12A—C11—O3—C10	158.9 (8)
C10—C7—C8—O2	−1.4 (8)	C12B—C11—O3—C10	−144.3 (13)
C1—C7—C8—O2	179.3 (6)		

### Hydrogen-bond geometry (Å, °)

**Table:**

Cg1 is the centroid of the C1–C6 benzene ring.

**Table:**

*D*—H···*A*	*D*—H	H···*A*	*D*···*A*	*D*—H···*A*
C2—H2···O1	0.93	2.35	3.018 (10)	129
C6—H6···O3	0.93	2.25	2.916 (8)	128
C9—H9B···Cg1^i^	0.97	2.80	3.632 (9)	144

Symmetry codes: (i) −*x*, −*y*+1, −*z*+1.
